# In vitro response of date palm (*Phoenix dactylifera* L.) to K/Na ratio under saline conditions

**DOI:** 10.1186/s40659-015-0055-2

**Published:** 2015-11-11

**Authors:** Suliman A. Alkhateeb, Abdullatif A. Alkhateeb, Mohei EL-Din Solliman

**Affiliations:** Environment and Natural Resources Department, College of Agriculture and Food Sciences, King Faisal University, P.O. Box 400, Hofuf, Alhassa 31982 Kingdom of Saudi Arabia; Agriculture Biotechnology Department, College of Agriculture and Food Sciences, King Faisal University, P.O. Box 400, Hofuf, Alhassa 31982 Kingdom of Saudi Arabia; Plant Biotechnology Department, National Research Centre, Dokki, 12622 Cairo, Arab Republic of Egypt

**Keywords:** Date palm, In vitro, Ion relations, K/Na ratio, Salinity

## Abstract

**Background:**

Salinity is a serious factor limiting the productivity of agricultural plants. One of the potential problems for plants growing under saline conditions is the inability to up take enough K^+^. The addition of K^+^ may considerably improve the salt tolerance of plants grown under salinity. It is assumed that increasing the K^+^ supply at the root zone can ameliorate the reduction in growth imposed by high salinity. The present study aims to determine whether an increase in the K/Na ratio in the external media would enhance the growth of date palm seedlings under in vitro saline conditions.

**Methods:**

Date palm plants were grown at four concentrations of Na + K/Cl (mol/m^3^) with three different K/Na ratios. The 12 salt treatments were added to modified MS medium. The modified MS medium was further supplemented with sucrose at 30 g/l.

**Results:**

Growth decreased substantially with increasing salinity. Growth expressed as shoot and root weight, enhanced significantly with certain K/Na ratios, and higher weight was maintained in the presence of equal K and Na. It is the leaf length, leaf thickness and root thickness that had significant contribution on total dry weight. Na^+^ contents in leaf and root increased significantly increased with increasing salinity but substantial decreases in Na^+^ contents were observed in the leaf and root with certain K/Na ratios. This could be attributed to the presence of a high K^+^ concentration in the media. The internal Na^+^ concentration was higher in the roots in all treatments, which might indicate a mechanism excluding Na^+^ from the leaves and its retention in the roots. K/Na ratios up to one significantly increased the leaf and root K^+^ concentration, and it was most pronounced in leaves. The K^+^ contents in leaf and root was not proportional to the K^+^ increase in the media, showing a high affinity for K^+^ uptake at lower external K^+^ concentrations, but this mechanism continues to operate even with high external Na^+^ concentrations.

**Conclusion:**

Increasing K/Na ratios in the growing media of date plam significantly reduced the absorption of Na^+^ less than 200 mM and also balance ions compartmentalization.

## Background

Salinity is a serious factor limiting the productivity of agricultural crops [20]. Although drainage and the supply of high-quality water can solve this problem, these measures are extremely costly and not feasible for extensive application to agriculture [25]. High salinity adversely affects plants due to water stress, ion toxicity, nutritional disorders, membrane disorganization, reduction of cell division and expansion [19, 28].

One of the potential problem for plants growing under saline conditions is the inability to uptake enough K^+^, thus creating K^+^ deficiency as a result of high concentration of Na^+^ and its competition with K^+^ [28]. Low uptake of K^+^ may occurs because, in saline soils, K^+^ is usually present at lower concentrations than Na^+^. Under non-saline conditions, plants are able to limit their Na^+^ uptake. However, with increasing salinity, Na^+^ is abundantly absorbed by most plants, even to lethal levels [20, 19]. The addition of K^+^ may considerably improve the salt tolerance of many crops [15, 32] by maintaining the ion transport balance across the plasma- and intra-organelle membrane [10, 27]. Application of Multi-K (potassium nitrate) is a very efficient method of combating stresses and enhancing crop performance under saline conditions. This concept has been validated for five crops [1].

It is generally assumed that Na^+^ is compartmentalized into vacuole, unlike K^+^, which is sequestered in the cytoplasm, resulting in maintenance of a high K/Na ratio in cytoplasm [17, 24]. However, Cuin et al. [8] in quantitative measurements of cytosolic potassium activity in leaf cell compartments of plants subjected to salt stress, reported reduced cytosolic potassium activity to 15 mM despite the vacuole still contain 47 mM. Potassium is ultimately involved in mitigating the detrimental effects of salinity to plant metabolism [29]. Very recently Anschutz et al. [6] in their review showed that regulation of intercellular potassium homeostasis is essential to mediate plant response to a broad range of biotic stresses including drought, salinity and oxidative stress.

 An inadequate rate of accumulation of osmotic solute in growing tissues of roots and leaves may limit the growth of many crop plants. High Na^+^ concentrations in the leaves may help to maintain turgor, which drives growth in the growing zone [31], but it cannot substitute for adequate K^+^ concentration, presumably because K^+^ plays essential roles in energy transfer and utilization, protein synthesis, carbohydrate metabolism, transport of sugars from leaves to fruits, and production and accumulation of oils [23]. However, high accumulation of Na^+^ in plant tissue may cause damaging effects due to ion excess. Moreover, the ability of the plant to maintain high cytosolic K/Na ratio has been named as a key determinator of a plant salt tolerance [18, 28].

Date palm (*Phoenix dactylifera* L.), being cultivated mostly in arid and saline conditions, and affected by excess salts, has not been previously explored for most of the physiological responses such as K^+^/Na^+^ ratio. The present studies were therefore, aimed to determine whether an increase in the K/Na ratios in the external media would enhance the growth of date palm seedlings under in vitro saline conditions.

## Results

Growth expressed as root, shoot and total dry weights reduced substantially with 200 mol/m^3^ (Na + K)/Cl compared to 10 mol/m^3^ (Na + K)/Cl (Table 1). Adverse effects of increasing (Na + K)/Cl concentration were more pronounced on shoots than on roots. Root and shoot dry weights of date palm seedlings were drastically increased in the presence of equal concentrations of K^+^ and Na^+^, even at 200 mol/m^3^ (Na + K)/Cl (Table 1). The shoot/root ratio significantly reduced at the highest salinity level (Table 1). However, it was significantly increased with increasing K/Na ratios from 0–1.

**Table 1:** Effects of Na + K/Cl concentration and K/Na ratio on leaf and root dry weight (mg/plant), total dry weight (mg/plant) and leaf dry weight/root dry weight ratio

Interaction (salinity × K/Na ratio)	Leaf dry weight	Root dry weight	Total dry weight	Ratio of leaf d.w./root d.w.
10 mol/m^3^				
0	268	125	393	2.329
0.5	428	134	562	3.953
1	359	208	567	1.845
50 mol/m^3^				
0	267	132	399	2.923
0.5	262	165	427	1.742
1	338	133	471	3.107
100 mol/m^3^				
0	408	143	551	2.875
0.5	351	113	465	3.821
1	393	152	545	2.676
200 mol/m^3^				
0	180	135	315	1.591
0.5	224	179	403	1.272
1	281	137	418	2.585
SEDs (d.f.)	51 (6)	11.5 (6)	48.3 (6)	0.46 (6)

Stander error deviation (SEDs) at P < 0.05

**Fig. 1:**
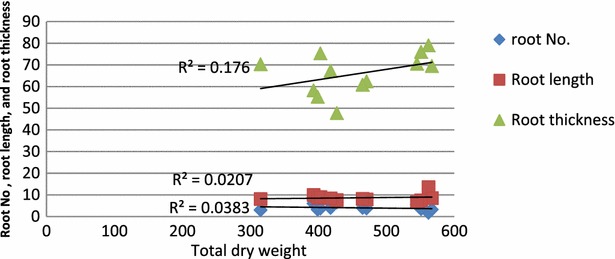
Relationship between root no., root length (mM) and root thickness (µM) and total dry weight (mg/plant) of date palm as affected by salinity and K/Na ratio

**Fig. 2:**
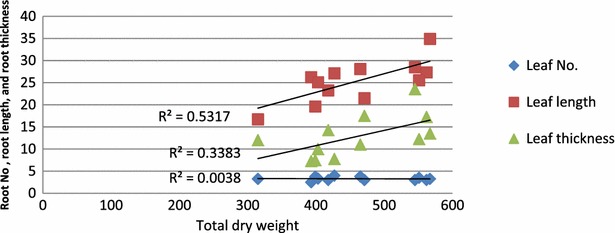
Relationship between root no., root length (mM) and root thickness (µM) and total dry weight (mg/plant) of date palm as affected by salinity and K/Na ratio

Regression between root thickness and total dry weight gave nonlinear correlations but with relatively good to fair coefficient of determination r^2^ = 0.45 while coefficient of determinations r^2^ of root number and root length were very poor (Table 2; Fig. 1). On the other hand Regression between leaf length, and leaf thickness and total dry weight gave nonlinear correlations but with relatively good to fair coefficient of determinations r^2^ = 0.55 and 0.37 respectively, while coefficient of determination r^2^ of leaf no. was very poor (Table 2; Fig. 2).

**Table 2:** Effects of Na + K/Cl concentration and K/Na ratio on root and leaf number, root and leaf length (mM), root and leaf thickness (µM)

Interaction (salinity × K/Na ratio)	Root no.	Leaf no.	Root length	Leaf length	Root thickness	Leaf thickness
10 mol/m^3^						
0	6.00	2.50	9.96	26.22	58.26	7.26
0.5	1.76	3.00	13.58	27.32	79.00	17.26
1	3.26	3.26	8.500	34.88	69.50	13.50
50 mol/m^3^						
0	3.26	3.76	8.56	19.62	55.26	7.48
0.5	6.50	4.00	7.62	27.12	47.76	7.76
1	3.76	3.00	7.92	21.50	62.40	17.50
100 mol/m^3^						
0	3.50	3.50	7.48	25.56	76.00	12.26
0.5	4.00	3.80	8.16	28.08	60.80	11.00
1	5.50	3.00	6.68	28.50	70.50	23.50
200 mol/m^3^						
0	3.00	3.26	8.10	16.72	70.26	12.00
0.5	3.68	3.32	9.00	25.10	75.32	10.00
1	4.00	3.00	8.38	23.26	67.26	14.26
SEDs (d.f)	0.365 (6)	0.352 (6)	1.21 (6)	2.89 (6)	10.5 (6)	1.76 (6)

Stander error deviation (SEDs) at P < 0.05

**Fig. 3:**
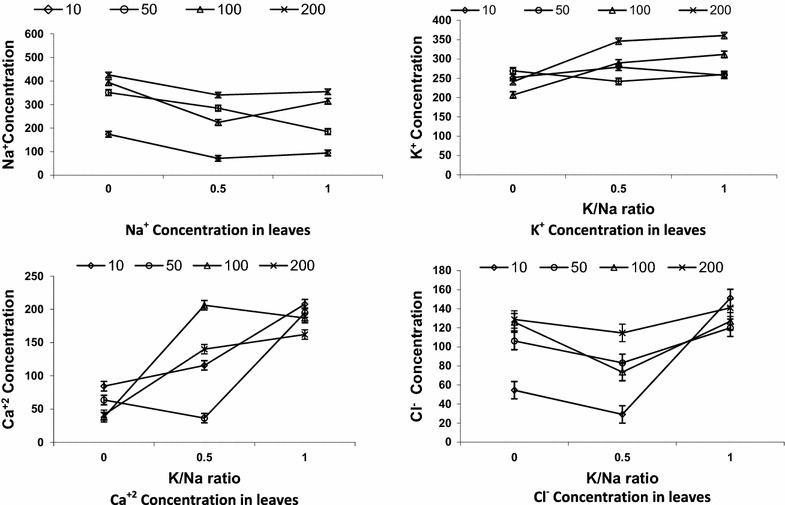
Na^+^, K^+^, Ca^+2^ and Cl^−^ concentrations in date palm leaves as affected by the interaction of (Na + K)/Cl concentration (mol/m^3^) and KNa ratio. *Error bars* are SEDs, d.f. = 6

**Fig. 4:**
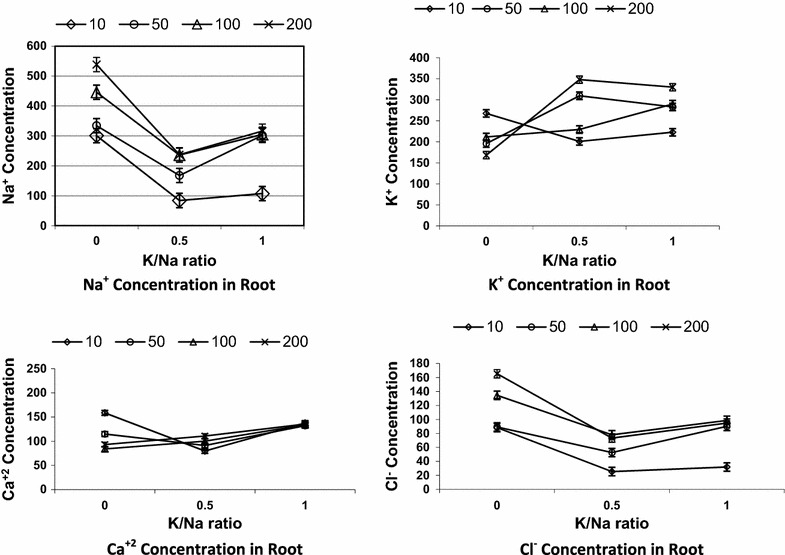
Na^+^, K^+^, Ca^+2^ and Cl^−^ concentrations in date palm root as affected by the interaction of (Na + K)/Cl concentrations (mol/m^3^) and K/Na ratio. *Error bars* are SEDs, d.f. = 6

The Na^+^ content increased significantly as the (Na + K)/Cl concentration increased, particularly at the 0 K/Na ratio (Fig. 3). However, at 200 mol/m^3^ (Na + K)/Cl, the leaf Na^+^ concentration decreased significantly at both the 0.5 and 1 K/Na ratios (Fig. 3). Varying K/Na ratios from 0–1 consistently decreased the Na^+^ concentration in roots and leaves at all (Na + K)/Cl concentrations. The only exception was observed in leaves at 10 mol/m3 (Na + K)/Cl, for which K/Na at the 0.5 ratio had the highest Na^+^ concentration (Fig. 3). The K^+^ contents in leaves and roots decreased significantly as the (Na + K)/Cl concentration increased (Fig. 4). K/Na ratios up to 1 increased significantly the K^+^ concentration in leaves and roots, and it was especially pronounced in roots at higher K/Na ratios (Fig. 4).

The root Ca^2+^ concentration of 10 mol/m^3^ (Na + K)/Cl was highest at the K/Na ratios of 0.5 and 1. The root Ca^2+^ concentration was significantly reduced at the K/Na ratio of 1.0 at the highest (Na + K)/Cl concentrations (Fig. 4). At 10 mol/m^3^ (Na + K)/Cl, the Ca^2+^ concentration in the leaves was significantly increased with increasing K/Na ratios. However, at 100 mol/m^3^ (Na + K)/Cl, the Ca^2+^ concentration in the leaves remained relatively unchanged with varying K/Na ratios, and it was lower than in the 10 and 50 mol/m^3^ (Na + K)/Cl conditions (Fig. 3).

Root Cl^−^ concentration was significantly increased as Cl^−^ concentration increased in the root media (Fig. 4). Varying K/Na ratios had no significant effects on root Cl^−^ concentrations. The Cl^−^ concentration followed approximately the same patterns in leaves and in roots (Figs. 3, 4).

## Discussion

**Fig. 5:**
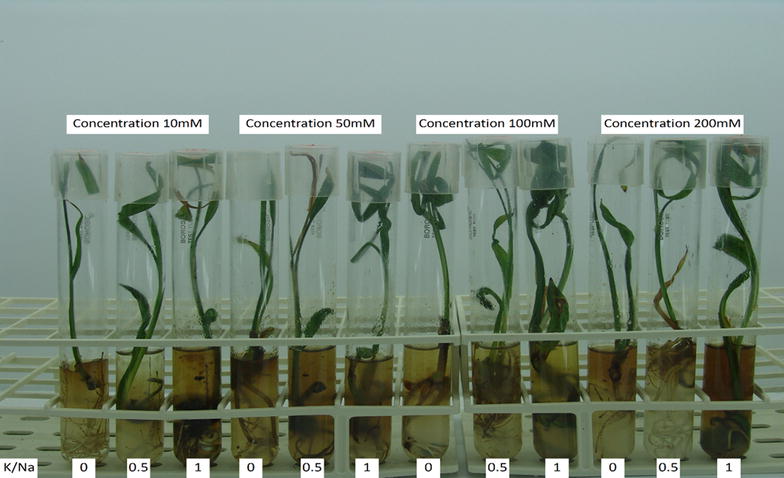
Effects of salinity and K/Na ratio on date palm cv. Barhi seedlings in vitro

Growth expressed as root, leaf and total dry weights reduced substantially in the presence of 200 mol/m^3^ (Na + K)/Cl (Table 1). This reduction under salinity is consistent with the results of Aljuburi et al. [3], Al-Abdoulhadi et al. [2], Darwesh [9] and Sperling et al. [30] in date palm. High NaCl levels inhibited leaf expansion, largely due to an inhibition of cell division rather than of cell expansion [7]. Adverse effects of increasing (Na + K)/Cl concentration were more pronounced in leaves than in roots, indicating that root growth was less affected by salinity; this was supported by the results for root and leaf length (Table 2). Consequently, the leaf/root ratio was expected to decrease with increasing (Na + K)/Cl concentrations. Moreover, visual observation (Fig. 5) of date palm offshoots grown under the highest salinity concentration showed necrosis at the tips and margins of leaves, which could be attributed to salt toxicity.

The increase in root and shoot dry weights of seedlings at K/Na ratio of 1, even at the highest salinity level (Table 1) is consistent with the results of Achilea [1] in five crop species. Concerning the relationship of total dry weight and growth parameters, the relatively good to fair correlations indicated that leaf length, leaf thickness, and root thickness had significant contribution in total dry weight (Figs. 1, 2). Shoot/root ratio reduced significantly at high salinity, but increased substantially with increasing K/Na ratios from 0–1. This might be due to dry weight partitioning between shoot and root, being significantly affected by varying K/Na ratios, in addition, root growth was always less affected by salinity than shoot [24].

Increasing leaf and root internal Na^+^ concentrations with increasing salinity levels, particularly at lower K/Na ratio (Figs. 3, 4), were reported in date palm [2, 3, 9, 30]. However, at the highest (Na + K)/Cl concentration, the leaf Na^+^ concentration decreased significantly at both 0.5 and 1 K/Na ratios (Fig. 3). This could be attributed to the presence of a high concentration of K^+^ in the media. The internal Na^+^ concentration was higher in the roots than leaves with all treatments, which might indicate a mechanism that excluded Na^+^ from the leaves and caused its retention in the roots. Such a mechanism helps to maintain the level of Na^+^ in leaves at low concentration. However, to protect metabolism from adverse effects of high Na^+^ concentrations in roots or leaves, ion compartmentation is required to take place in the different cell components. Leigh and Wyn Jones [16] reported that Na^+^ ions often excluded from the cytoplasm and accumulated in vacuoles. Varying K/Na ratios decreased Na^+^ concentrations in roots and leaves at all (Na + K)/Cl concentrations. The lower Na^+^ concentrations in leaves and roots of plants grown with K/Na ratios of 1 could be attributed to the slower growth of these plants, particularly for the leaves. Alternatively, the rate of exporting Na^+^ from roots to leaves may have been lower in plants grown with a K/Na ratio of 1 than in those grown with K/Na ratios lower than 1.

The K^+^ contents in leaves and roots decreased significantly with increase in Na + K/Cl concentration in the growing medium (Figs. 3, 4). The lower internal K^+^ contents with an increase in external Na^+^ concentration in the absence of K^+^ in date palm might be due to the tendency of the Na^+^ to compete with K^+^ for major binding sites including control of enzymatic activity or a direct competition between K^+^ and Na^+^ [29]. K/Na ratios up to 1 significantly increased leaf and root K^+^ contents with substantial increase in leaves particularly at higher K/Na ratios (Figs. 3, 4). However, there was no proportionality of leaf and root K^+^ contents to K^+^ increase in the external media, showing a high affinity for K^+^ uptake at low external K^+^ concentration, but this mechanism is still operative even with high external Na^+^ concentrations [11]. K^+^ contents were higher in roots than in leaves with the absence of salinity. This was expected because K^+^ and Na^+^ contents in the roots depend on active and passive fluxes of Na^+^ and K^+^, respectively, into and out of the root.

The date palm seedlings grown at 100 mol/m^3^ Na + K/Cl with a K/Na ratio of 0 and 1, had 206 and 210, 290 and 310 mol/m^3^ K^+^ in roots and leaves, respectively (Figs. 3, 4). However, to protect the plant metabolism from excess ions, compartmentalization is required. [8] reported unequal compartmentation of K^+^ between cytosol and vacuole, with a threefold more K^+^ in vacuole than cytosol. With this assumption, cytoplasm of root and leaf will have around 70 and 100 mol/m^3^ K^+^ in a K/Na ratio of 0 and 1, respectively. According to He and Wang [14], seedlings grown under saline conditions accumulated more than 60 % of Na^+^ in vacuoles. Assuming this, Na^+^ contents in the cytoplasm of roots and leaves will be approximately 178 and 157 mol/m^3^, 122, and 125 with 0 and 1 K/Na ratios, respectively. Groham et al. [13] reported that the concentration of inorganic ions in the cytoplasm (especially of meristematic cells) is in the range of 100–200 mol/m^3^. This situation reflects a low internal K/Na contents in cytoplasm particularly under high salinity with the absence of K^+^ supply. Alternatively, ions may accumulate in the cell wall reducing turgor pressure [22], which is the driving force of plant growth. Moreover, the regulation of intercellular potassium homeostasis is also essential to mediate plant response to a broad range of biotic stresses including drought, salinity and oxidative stress [6]. Moreover it is suggested that not only cytosolic K/Na ratios but also absolute concentrations of K^+^ are essential for conferring salinity stress tolerance [29].

Decrease in Ca^2+^ concentration in roots and leaves of date palm with increasing (Na + K)/Cl concentrations might have induced displacement of Ca^2+^ by Na^+^ in cell membrane [11]. However, Darwesh [9] elucidated that date palm plantlets clarified high calcium contents under salinity applications. The removal of Ca^2+^ from the membrane affects adversely the mechanism of selective ion transport and increase membrane permeability [5]. However, it appears that the presence of K^+^ in the external media positively affects Na^+^ displacement of Ca^2+^, particularly with the highest (Na + K)/Cl concentrations.

Root Cl^−^ concentration increased significantly as Cl^−^ concentration increased in the root media (Fig. 4). Darwesh [9] obtained similar results growing date palm seedlings under salinity and amino acid treatment. It appears that varying K/Na ratios had no significant effects on root Cl^−^ concentrations. Roots had much higher Cl^−^ concentrations than leaves. Leaf Cl^−^ concentrations followed approximately the same pattern as in root (Figs. 3, 4). Groham et al. [13] showed that ion toxicity was usually associated with either excessive chloride or sodium intake. However, there was a slight increase in the leaf Cl^−^ concentration with increasing K/Na ratios. Moreover, Cl^−^ is a prevalent anion accompanying K^+^ or Na^+^; therefore, its concentration is expected to be equivalent to the sum of Na + K. This concurrence of Na + K complicates the evaluation of Cl^−^ specific toxicity. At 200 mol/m^3^ (Na + K)/Cl, the internal Na + K concentration in roots and leaves were much larger than Cl^−^ concentration. Although Na^+^ appears to reach a toxic concentration before Cl^−^ for most species, while for other species such as soybean, citrus and grapevine, Cl^−^ is regarded as more toxic than Na^+^ [24].

## Conclusion

The growth of date palm reduced substantially with increasing salinity. Increasing K/Na ratio on the growing media enhanced date palm seedling growth. This improvement in growth was accompanied by a decrease in Na^+^ concentration and an increase in K^+^ concentration in the plant tissue with lower Cl^−^ concentrations in leaves and roots of date palm. Adding K^+^ to the salt containing media of date palm reduced the absorption of Na^+^ less than 200 mM and also balances ions compartmentalization.

## Methods

This experiment was conducted in the Tissue Culture Laboratory of the College of Agriculture and Food Sciences, King Faisal University, Kingdom of Saudi Arabia. Date palm offshoots cv. Barhi of approximately 3 to 4 years old and weighing 5–7 kg were separated from healthy mother trees. Offshoots were cleaned thoroughly and the outer leaves were carefully removed to expose the region of the shoot tip and lateral buds. The exposed region was excised and placed immediately in antioxidant solution containing 15 mg/l ascorbic acid and 100 mg/l citric acid. The shoot tip and lateral buds were sterilized in 20 % v/v Clorox solution for 15 min, followed by rinsing 3 times with distilled water. The tissues were kept in the previous antioxidant solution until explant excision for culturing. The shoot tip and lateral buds were sectioned into explants of approximately 1 cm, which were used for culture initiation as described by Alkhateeb and Ali-Dinar [4]. One rooted plant resulted from rooting media was transferred to a test tube of 20 mm in diameter and 200 mm in length filled with 15 ml of modified MS salts medium [21] supplemented with 125 mg/l inositol; 200 mg/l glutamine; 1 mg/l thiamine HCl; 1 mg/l pyridoxine HCl; 1 mg/l nicotinic acid; 1 mg/l calcium pantothenate; 1 mg/l biotin; 7 g/l purified agar, and 30 g/l sucrose. Potassium phosphate, potassium nitrate, potassium iodide, sodium molybdate, and Na2EDTA·2H_2_O were eliminated from the MS media to avoid any interference with the treatment concentrations of Na and K. The modified MS media was supplemented with the 12 salt treatments (Table 3). Cultures were incubated at 25 ± 2 °C with 16 h of light daily supplied by 65/80 Warm White Weisse 3500 fluorescent tubes. Each treatment was represented by 10 replicates (tubes) in a factorial, completely randomized design.

**Table 3:** Concentration of K^+^ and Na^+^ (mol/m^3^) required for the evaluation of K/Na ratio in the external media

(Na + K) Cl concentration (mol/m^3^)	K/Na ratio		
	0.0	0.5	1.0
10			
K^+^	0	3.33	5
Na^+^	10	6.67	5
50			
K^+^	0	16.7	25
Na^+^	50	33.3	25
100			
K^+^	0	33.3	50
Na^+^	100	66.7	50
200			
K^+^	0	66.7	100
Na^+^	200	133.3	100

(K and N) were supplied as KCl and NaCl, respectively

Plants were harvested 3 months after the treatments were applied. Plants were separated into shoot and roots, and their fresh weights were determined. The shoots were washed twice in distilled water, and ions were removed from the free spaces around roots by washing for 2 min in sorbitol solutions isotonic with the treatment concentration in which the plants had grown. To determine the dry weight, shoots and roots were dried at 85 °C for 48 h. For the analysis of K^+^, Na^+^, Ca^2+^, and Cl^−^, samples of 500 mg of fresh material of leaves or roots were homogenized using a mortar and pestle and were extracted in 25 ml of distilled deionized water at 90 °C for 4 h. The Na^+^ and K^+^ contents were determined with a flame photometer (Jenway, PFP7). Ca^2+^ was measured with a GBS 905 atomic absorption spectrophotometer. Cl^−^ was determined using a chloride meter (Jenway, PCLLM3).

Data was subjected to statistical analysis as a factorial design according to Gomez and Gomez [12]. Statistical analyses were performed using SAS software [26]. Means were separated by standard error deviation with their corresponding degrees of freedom.
